# Effects of ice slurry ingestion on body temperature and softball pitching performance in a hot environment: a randomized crossover trial

**DOI:** 10.1186/s40101-023-00329-0

**Published:** 2023-06-29

**Authors:** Urara Numata, Takuma Yanaoka, Shiho Kurosaka, Hiroshi Hasegawa

**Affiliations:** Graduate School of Humanities and Social Sciences, Hiroshima University, 1-1-1 Kagamiyama, Higashi-Hiroshima, Hiroshima, 739-8524 Japan

**Keywords:** Ball velocity, Body cooling, Body temperature, Heat, Muscle strength, Pitching accuracy, Pre-cooling

## Abstract

**Background:**

Although softball players are often required to play in hot environments, scarce evidence is available regarding the effects of ice slurry ingestion on body temperature and pitching performance in softball pitchers in a hot environment. Thus, this study investigated the effects of ice slurry ingestion before and between innings on body temperature and softball pitching performance in a hot environment.

**Methods:**

In a randomized crossover design, seven heat-acclimatized amateur softball pitchers (four males and three females) completed simulated softball games consisting of 15 best-effort pitches per inning for seven innings with between-pitch rest intervals of 20 s. Participants were assigned to either a control trial (CON: ingestion of 5.0 g·kg^−1^ of cool fluid [9.8 ± 2.2 °C] before simulated softball games and 1.25 g·kg^−1^ of cool fluid between inning intervals) or an ice trial (ICE: ingestion of ice slurry [− 1.2 ± 0.1 °C] based on the same timings and doses as the CON). Participants performed both trials in an outdoor ground during the summer season (30.8 ± 2.7 °C, 57.0 ± 7.9% relative humidity).

**Results:**

Ice slurry ingestion before the simulated softball game (pre-cooling) resulted in a greater reduction in rectal temperature compared with cool fluid ingestion (*p* = 0.021, *d* = 0.68). No significant differences were observed between the trials in rectal temperature changes during the simulated softball game (*p* > 0.05). Compared to the CON, heart rate during the game was significantly decreased (*p* < 0.001, *d* = 0.43), and handgrip strength during the game was significantly increased (*p* = 0.001, *d* = 1.16) in the ICE. Ratings of perceived exertion, thermal comfort, and thermal sensation were improved in the ICE compared to those in the CON (*p* < 0.05). Ball velocity and pitching accuracy were not affected by ICE.

**Conclusions:**

Ice slurry ingestion before and between innings reduced thermal, cardiovascular, and perceptual strain. However, it did not affect softball pitching performance compared to cool fluid ingestion.

**Supplementary Information:**

The online version contains supplementary material available at 10.1186/s40101-023-00329-0.

## Background

The pitcher is one of the most important positions in softball, and ball velocity is an important indicator of performance in softball pitchers. However, ball velocity generally decreases with the increase in number of pitches thrown [1]. Possible mechanisms underlying decreased ball velocity for softball pitchers include increased pain, reduced muscle strength, and decreased flexibility [2]. A crossover effect of muscle fatigue from exercised to nonexercised upper extremity muscles has also been reported [1]. These findings suggest that the decrease in ball velocity with the increase in number of pitches thrown may result from central fatigue (e.g., the ability to voluntarily activate motor units) as well as peripheral fatigue.

Although softball players are often required to play in hot environments, little evidence is available regarding the magnitude of increases in body temperature and decreases in pitching performance during softball games played in hot environments. Exercising in a hot environment, compared with a temperate environment, results in a faster rise in body core (*T*_c_) and mean skin ($\overline{T }$_sk_) temperatures [3]. In a hot environment, a concurrent rise in *T*_c_ and $\overline{T }$_sk_ can lead to greater cardiovascular, metabolic, and perceptual strain, resulting in impaired exercise performance and an increased risk of heat-related illness [3]. Moreover, hyperthermia-induced central fatigue downregulates skeletal muscle recruitment and decreases the maximal voluntary isometric contraction [3]. Previous studies have reported that the tympanic temperature in heat-acclimatized baseball players reached 39 °C or higher after a 3-h practice session in a hot environment [4]. The gastrointestinal temperature in bowlers increased to 38.8 °C during bowling simulations in cricket matches [5]. In addition, Huang et al. reported that an extremely hot environment resulted in decreased pitching performance, although this did not affect hitting performance in the Chinese Professional Baseball League games in Taiwan [6]. Accordingly, it can be assumed that in a hot environment, *T*_c_ in the softball pitcher increases significantly with an increase in the number of pitches thrown, resulting in a greater increase in the magnitude of ball velocity decrement. Therefore, it is important to develop practical strategies for aggressive cooling in softball pitchers.

Cooling the body may be an effective approach as this strategy can alleviate physiological (elevated T_c_, $\overline{T }$_sk_, and heart rate [HR]) and perceptual (increased rate of perceived exertion [RPE], thermal sensation [TS], and thermal comfort [TC]) strain in a hot environment [3, 7]. Body cooling strategies may be classified as internal and external cooling strategies. Several studies have investigated the efficacy of external cooling strategies for baseball and cricket players [5, 8–11]. External cooling of cricket pitchers was shown to prevent increases in *T*_c_, $\overline{T }$_sk_, HR, RPE, and TS during bowling simulations in a hot environment [5]. In another study, external cooling of baseball pitchers prevented decreases in ball velocity during simulated baseball games in a thermoneutral environment [10]. However, there is currently no evidence regarding the efficacy of internal cooling strategies on pitching performance. Ingestion of ice slurry before exercise can effectively lower *T*_c_ compared with the ingestion of cool fluid [12]. Previous studies have suggested that ice slurry ingestion may improve hyperthermia-induced central fatigue by decreasing brain temperature [13] and by cold stimulation of internal thermoreceptors in the mouth, throat, and stomach regions [14]. Moreover, repeat ingestion of ice slurry during breaks in a tennis-simulated game was found to increase its cooling power [15, 16]. Therefore, ice slurry ingestion before and between innings could improve physiological and thermal strain as well as pitching performance in softball pitchers.

Although evidence suggests an improvement in physiological and thermal strain following ice slurry ingestion, the efficacy of this strategy can be affected by the heat-acclimatization status of the players [17] and no wind velocity and solar radiation due to laboratory settings [18]. Moreover, many studies used an ambient temperature fluid as a control condition, although athletes often ingest cold fluid during sports competitions [19]. These methodological limitations suggest that previous findings may have overestimated the physiological and ergogenic benefits of ice slurry ingestion. Therefore, this study aimed to investigate the effects of ice slurry ingestion before and between innings on body temperature and softball pitching performance in a hot environment. This study included heat-acclimatized softball pitchers, simulated softball games in an outdoor ground, and used a cool fluid (10 °C) as a control condition. We hypothesized that ice slurry ingestion before and between innings would decrease thermal (*T*_c_), cardiovascular (HR), and perceptual strain (TS, TC, and RPE) and attenuate the decrease in ball velocity that occurs with the increase in the number of pitches thrown.

## Methods

### Participants

A sample size of seven participants was required based on a power calculation (G*Power 3) [20], with an *α* of 0.05, a *β* of 0.20, and an effect size (Cohen’s d) of 1.43, calculated from the difference in *T*_c_ following repeated ice slurry ingestion demonstrated in a previous study [16]. Seven heat-acclimatized softball pitchers participated in this study (four male participants: age, 22.8 ± 1.8 years; height, 1.69 ± 0.04 m; body mass, 65.7 ± 6.1 kg; three female participants: age, 22.0 ± 0.8 years; height, 1.60 ± 0.05 m; and body mass, 58.6 ± 4.5 kg [mean ± standard deviation]). All participants were amateur softball pitchers with no history of elbow or shoulder injury within the 6 months before the study. The female participants experienced regular menstrual cycles and did not use oral contraceptives. The participants dressed in a softball attire, consisting of a cap, softball pants, socks, softball shoes, practice jerseys, and belts. This study was approved by the Ethics in Human Research Committee of the Graduate School of Humanities and Social Sciences at Hiroshima University (approval number: 2021074). All participants provided written informed consent to participate in this study.

### Experimental design

All participants completed two experimental trials using a randomized crossover design in an outdoor softball ground. The trials were separated by at least 7 days and were performed at the same time of the day (09:30–15:00) for each participant to avoid any circadian rhythm-related variations. Female participants were tested between days 2 and 10 of their menstrual cycle. Participants refrained from consuming alcohol and caffeine for 24 h prior to each experimental trial and fasted for 2 h, excluding the consumption of water, before each experimental trial. The participants were asked to avoid altering their regular lifestyle habits, exercise, and diet throughout the study. This study was completed during the summer season (30.8 ± 2.7 °C, 57.0 ± 7.9% relative humidity, 1.6 ± 0.8 m/s for air velocity).

### Simulated softball games

**Fig. 1:**
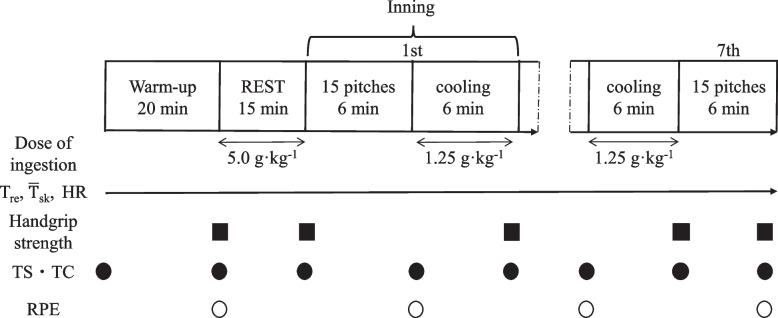
Schematic representation of the experimental protocol. *T*_re_, rectal temperature; $\overline{T }$_sk_, mean skin temperature; TS, thermal sensation; TC, thermal comfort; RPE, rating of perceived exertion

Figure 1 demonstrates the testing protocol. Upon arrival, the participants first emptied their bladders. Nude body mass and urine-specific gravity were then measured in all participants. After that, the participants were then fitted with the measuring instruments. After the standardized warm-up (2-min jogging, 5-min active stretching, and 13-min practice throwing, with gradually increasing ball velocity), participants sat in a dugout for 15 min (REST). After REST, participants threw 15 best-effort pitches per inning to a catcher for seven innings, with between-pitch rest intervals of 20 s [21]. A 6-min interval was allowed between the innings. The participants were instructed to throw fastballs into the strike zone. Three practice pitches were allowed prior to each inning. For the best-effort pitches, the batter was made to stand.

### Cooling intervention

Participants were assigned to either a control trial (CON: ingestion of 5.0 g·kg^−1^ of fluid during REST and 1.25 g·kg^−1^ of fluid between the inning intervals; 9.8 ± 2.2 °C) or an ice trial (ICE: ingestion of 5.0 g·kg^−1^ of ice slurry during REST and 1.25 g·kg^−1^ of ice slurry between the inning intervals; − 1.2 ± 0.1 °C). The participants undertook both interventions in a dugout. The ice slurry was made from conventional sports drinks (Pocari Sweat, Otsuka Pharmaceutical, Japan) containing 6.2 g of carbohydrates, 49 mg of sodium, and 20 mg of potassium per 100 mL. A commercially available ice shaver was used to create the ice flakes. The ice flakes and 4 °C conventional sports drink were mixed in a 3:1 ice-to-drink ratio to create an ice slurry. The fluid used in the CON had the same components as those in the ice slurry.

### Measurements

#### Physiological index

Rectal temperature (*T*_re_) was measured using a thermistor (LT-ST08-21, Nikkiso-Therm, Japan) at a depth of 10 cm from the anal sphincter at 1-min intervals. $\overline{T }$_sk_ was measured using a wireless surface temperature data logger (Thermochron SL, KN Laboratories, Japan) at 1-min intervals. The thermistor was attached to four sites (chest, upper arm, thigh, and calf). $\overline{T }$_sk_ was calculated as follows: $\overline{T }$_sk_ = 0.3 (chest + upper arm) + 0.2 (thigh + calf) [22]. For simplicity of the statistical analysis, body temperature data are presented as the 1-min sample measured immediately before and after warm-up and innings. Moreover, changes (*Δ*) in *T*_re_ during REST (i.e., 15-min rest between the post-warm-up and post-REST) and the simulated softball games (i.e., between the post-REST and seventh inning) were calculated.

HR was measured using a wireless HR monitor (RS800CX, Polar Electro, Finland) at 1-min intervals. The towel-dried nude body mass was measured using a scale (InBody 470, InBody Japan, Japan) before and after the experimental trial. Gross sweat loss was estimated by adjusting for fluid intake. The urine-specific gravity was measured using a refractometer (PAL-09S, Atago, Japan) before and after the experimental trial.

#### Perceptual indices

TS (− 6 [very cold] to 6 [very hot]) and TC (− 6 [very uncomfortable] to 6 [very comfortable]) were measured before and after warm-up and innings [23]. The RPE (6 [no exertion] to 20 [maximal exertion]) was assessed after each inning [24].

#### Pitching performance and muscle strength

The ball velocity was measured using a radar gun (Speedster V, Bushnell, USA) positioned 3 m behind the home plate. Video images were recorded using an HD 60 fps camera (GZ-E780, JVCKENWOOD, Japan) positioned 3 m behind the home plate. These video images were used to determine the pitching accuracy (i.e., the percentage of pitches crossing the strike zone during flight). Accuracy was determined by the same investigator. The handgrip strength of the dominant hand was measured using a handgrip dynamometer (T.K.K.5401, Takei, Japan) after the warm-up, REST, and each inning.

### Statistical analysis

Statistical analysis was performed using SPSS software (Version 29.0, SPSS Japan Inc., Japan). Statistical significance was set at *p* < 0.05. Unless otherwise stated, all values are presented as the mean ± standard deviation. The Shapiro–Wilk test was used to assess the normality of distribution, and all parameters, except for the perceptual indices, were found to not differ significantly from normal. Differences in environmental variables between trials were analyzed using linear mixed models (fixed effects: trial). Differences between trial, time, and trial × time for the changes in thermal, cardiovascular, and performance variables were analyzed using linear mixed models (fixed effects: trial and time; random effect: participant). This analysis was preferred because it allows for missing data. It also accurately models different covariate structures for repeated-measures data and models between-subject variability [25]. Changes (*Δ*) in *T*_re_ during REST and the simulated softball games were calculated and analyzed using linear mixed models to adjust for potential confounding covariates (i.e., *T*_re_ at the end of the warm-up or the commencement of the first inning and wet bulb globe temperature). Where significance was found, the values were subsequently analyzed using the Bonferroni multiple comparison test. Differences in perceptual variables between trials were analyzed using the Wilcoxon’s matched pairs test. Cohen’s d effect size was also calculated where necessary, whereby > 2.0 was categorized as a very large effect, 1.2–2.0 as a large effect, 0.6–1.2 as a moderate effect, and 0.2–0.6 as a small effect [26].

## Results

The mean data for all participants, male participants, and female participants are presented in Supplementary Tables 1, 2, and 3, respectively. No significant differences were observed in the ambient temperature (CON: 31.1 ± 3.9 °C, ICE: 30.5 ± 2.2 °C, *p* = 0.539), relative humidity (CON: 53.4 ± 12.7%, ICE: 60.6 ± 6.5%, *p* = 0.124), and air velocity (CON: 1.1 ± 0.9 m/s, ICE: 1.5 ± 1.3 m/s, *p* = 0.573) between trials.

### Body temperature

**Fig. 2:**
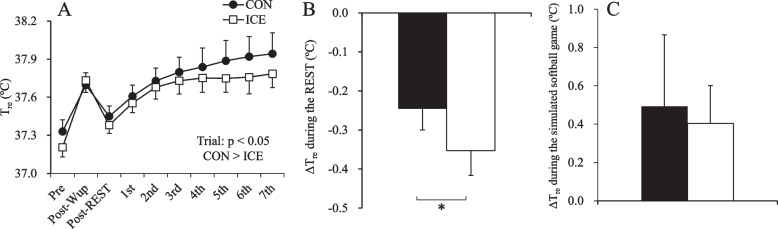
*T*_re_ (**A**) and *ΔT*_re_ during REST (**B**) and simulated softball games (**C**) between the trials. The values are shown as mean ± standard deviation (*n* = 7). *Significant difference between trials (*p* < 0.05). *T*_re_, rectal temperature; *ΔT*_re_, changes in rectal temperature

*T*_re_ responses are shown in Fig. 2A. No trial × time interactions were observed for *T*_re_ (*p* > 0.05) and $\overline{T }$_sk_ (*p* > 0.05, Supplementary Table 1). However, there was a main effect of the trial for *T*_re_ (*p* < 0.001). *T*_re_ was significantly lower in the ICE than in the CON (*p* < 0.001, *d* = 0.46).

*ΔT*_re_ during REST and simulated softball games are shown in Fig. 2B and C, respectively. The ICE resulted in a greater reduction in *T*_re_ during REST than the CON (*p* = 0.021, *d* = 0.68, Fig. 2B). No significant difference between the trials was observed in *ΔT*_re_ during the simulated softball games (*p* > 0.05, Fig. 2C).

### Heart rate and body fluid balance

There was no trial × time interaction for HR (*p* > 0.05), but a main effect of the trial for HR was observed (*p* < 0.001, Supplementary Table 1). HR was significantly lower in the ICE than in the CON (*p* < 0.001, *d* = 0.43). Changes in towel-dried nude body mass, gross sweat loss, and urine-specific gravity remained comparable between the trials (*p* > 0.05).

### Perceptual indices

**Fig. 3:**
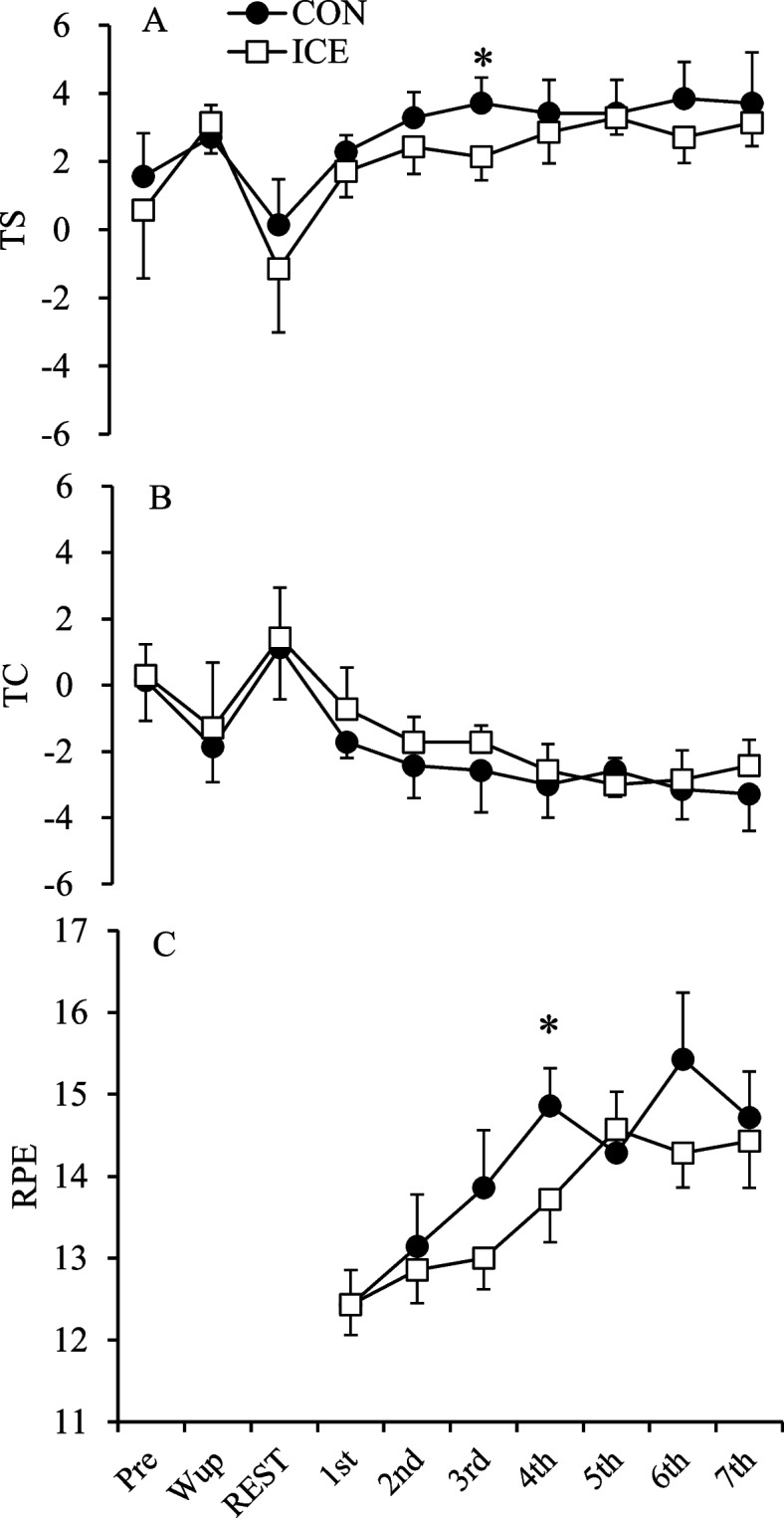
TS (**A**), TC (**B**), and RPE (**C**) between the trials. The values are shown as mean ± standard deviation (*n* = 7). *Significant difference between trials (*p* < 0.05). TS, thermal sensation; TC, thermal comfort; RPE, rating of perceived exertion

The perceptual indices between the trials are shown in Fig. 3. The ICE significantly lowered TS after the third inning (*p* = 0.026, *d* = 2.35) and RPE after the fourth inning (*p* = 0.023, *d* = 0.95). The ICE also lowered TS after the second inning (*p* = 0.063, *d* = 1.20) and TC after first (*p* = 0.059, *d* = 0.88), second (*p* = 0.059, *d* = 0.96), third (*p* = 0.058, *d* = 0.51), and seventh (*p* = 0.098, *d* = 0.96) innings; however, there were no significant differences between the trials.

### Pitching performance and muscle strength

**Fig. 4:**
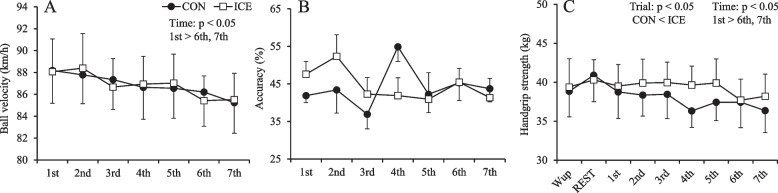
Ball velocity (**A**), accuracy (**B**), and handgrip strength (**C**) between the trials. The values are shown as mean ± standard error (*n* = 7)

The responses for ball velocity, pitching accuracy, and handgrip strength are shown in Fig. 4. There were no trial × time interactions for ball velocity (*p* > 0.05), pitching accuracy (*p* > 0.05), and handgrip strength (*p* > 0.05). However, main effects of time on ball velocity (*p* < 0.001) and handgrip strength (*p* = 0.003) were observed. The ball velocity was significantly lower in the sixth (*p* = 0.002, *d* = 0.33) and seventh (*p* < 0.001, *d* = 0.40) innings than in the first inning. Handgrip strength was significantly lower in the sixth (*p* = 0.009, *d* = 0.40) and seventh (*p* = 0.003, *d* = 0.44) inning than at REST. There was a main effect of the trial on handgrip strength (*p* = 0.001). Handgrip strength was significantly higher in the ICE than in the CON (*p* = 0.001, *d* = 1.16).

## Discussion

This study investigated the effect of ice slurry ingestion before and between innings on body temperature and softball pitching performance in a hot environment. The present findings revealed that, in accordance with our principal hypothesis, ice slurry ingestion before and between innings decreased thermal (*T*_re_), cardiovascular (HR), and perceptual (TS, TC, and RPE) strain. However, ice slurry ingestion had no impact on the magnitude of the ball velocity decrement with the increase in the number of pitches thrown.

To our knowledge, this study is the first to investigate the influence of ice slurry ingestion on *T*_c_ in softball pitchers playing in a hot environment. In the present study, the ingestion of 5.0 g·kg^−1^ of ice slurry during REST (i.e., pre-cooling) reduced *T*_re_ compared with the ingestion of 5.0 g·kg^−1^ of cool fluid (− 0.35 ± 0.16 °C vs − 0.25 ± 0.13 °C). A previous study reported a 0.4 °C reduction in *T*_re_ with 7.5 g·kg^−1^ of ice slurry ingestion for 15 min after warm-up [27], which is consistent with the present findings. Ice slurry ingestion as a pre-cooling strategy decreases *T*_re_ prior to exercise and increases heat storage capacity, which delays the influence of hyperthermia-induced fatigue [28]. However, this strategy also results in (1) a smaller core-to-skin temperature gradient, caused by a decrease in *T*_re_ without a concurrent decrease in $\overline{T }$_sk_, and (2) a delay in the onset of sweating during subsequent exercise [29]. These may lead to a greater rate of *T*_re_ increase during the early stages of subsequent exercise [29]. In the present study, the magnitude of the increase in *T*_re_ during simulated softball games was comparable between the trials (Fig. 2C), which is inconsistent with previous research [29]. This resulted from the ingestion of 1.25 g·kg^−1^ of ice slurry between the innings. A previous study reported that ingestion of 1.25 g·kg^−1^ of ice slurry in a game and set breaks during a tennis-simulated running test significantly lowered *T*_re_ compared with the ingestion of the same dose of cool fluid (4 °C) according to the same timing [15]. A high dose of ice slurry ingestion may result in gastrointestinal discomfort [30]. Therefore, the present results, which were obtained by employing an intermediate (5.0 g·kg^−1^) dose before simulated softball games and low (1.25 g·kg^−1^) doses between the innings of ice slurry ingestion, are useful for softball pitchers playing innings in a hot environment.

In the present study, neuromuscular function following ice slurry ingestion was assessed because reduced muscle strength was shown to be related to decreased ball velocity for softball pitchers [2]. Exercise-induced hyperthermia in a hot environment impairs neuromuscular function in both exercised and non-exercised muscle groups [31]. Impairment was found to be associated with central fatigue, which is triggered by an increase in *T*_c_ including brain temperature [3]. A previous study revealed that ingestion of 7.5 g·kg^−1^ ice slurry reduced brain temperature, as measured by proton magnetic resonance spectroscopy [13]. A previous study also demonstrated that ingestion of 1.25 g·kg^−1^ ice slurry attenuated reductions in maximal voluntary contraction following exercise-induced hyperthermia without causing physiological changes [14]. The authors postulated that the improvement in neuromuscular function resulted from the ice slurry by (1) altering afferent feedback by stimulating internal thermoreceptors in the mouth, esophagus, and stomach regions and (2) stimulating the reward/pleasure centers of the brain [14]. The present study demonstrated that ice slurry ingestion attenuated the reduction in handgrip strength compared with cool fluid ingestion. This result is consistent with the findings of a previous study [14]; therefore, the improvement may result from *T*_c_ reduction, including brain temperature, and sensory mechanisms.

The present study demonstrated that ice slurry ingestion lowered HR by approximately 10 bpm compared with cool fluid ingestion, which is consistent with the findings of a reduction in HR by ingestion of 1.25 g·kg^−1^ of ice slurry during game and set breaks in a tennis-simulated running test in a previous study [15]. We are unable to conclude the mechanism of reductions in HR with small decrements in *T*_re_ during the simulated softball game (i.e., approximately 0.1 °C) due to a lack of cardiovascular measurements (i.e., skin blood flow, stroke volume, cardiac output, and mean arterial pressure). However, given the results of a previous study [32], we speculate that this effect resulted from an enhancement of heat storage capacity by ingesting ice slurry. In support of this, Ng et al. reported that, compared to tepid fluid, ice slurry ingestion during exercise at 60% of maximal oxygen uptake decreased HR by 9 bpm without significantly changes in *T*_re_, skin blood flow, cardiac output, mean arterial pressure, or local sweat rate [32]. The authors suggested the possibility that ice slurry ingestion provided a sufficient heat sink to decrease heat storage and thereby reduce cardiovascular strain [32]. The other study also reported that ice slurry ingestion during walking at 4.0 km·h^−1^ on a 12% incline wearing firefighter-protective clothing decreased HR by approximately 10 bpm at 5 min of the commencement of walking compared to cold fluid [33]. However, this is only speculative, and further research is warranted to investigate the mechanism of the decrease in HR following ice slurry ingestion.

Although ice slurry ingestion improved thermal (*T*_re_), cardiovascular (HR), and perceptual (RPE, TS, and TC) strain, pitching performance was not affected by ice slurry ingestion. This may be due to the low thermal strain (i.e., low magnitude of the increases in *T*_re_) during the simulated softball games in this study. A recent review suggested that it is easier to avoid an increase in thermal strain by employing less intense exercise protocols or protocols interspersed with moments of lower-intensity recovery periods (intermittent efforts); thus, the benefits of ice slurry ingestion are minimized [34]. Indeed, the magnitudes of the increases in *T*_re_ during CON in the present study were lower than those observed in previous studies, which investigated changes in body temperature during a 3-h baseball practice [4] and bowling simulations in cricket matches played [5] in a hot environment. Therefore, future research is needed to investigate the ergogenic effect of ice slurry ingestion on pitching performance in environments with high thermal strain, such as actual softball games.

The strength of the present study includes its methodological setting: (1) trials were conducted in an outdoor ground, (2) pitchers were heat acclimatized, and (3) cool fluid (10 °C) was employed for CON. Previous studies were conducted in the laboratory; however, environmental factors, including wind velocity [18] and solar radiation [35], have a significant influence on body temperature during exercise. In addition, heat-acclimatized individuals may experience ceiling effects of body cooling strategies on thermal and performance variables [17]. Moreover, using an ambient-temperature fluid as a control condition may overestimate the extent of the physiologic and ergogenic benefits of ice slurry ingestion. Therefore, the present results, which were obtained using a method with high ecological validity, suggest that ice slurry ingestion is a physiologically effective heat mitigation strategy for softball pitchers.

This study has three limitations. First, the type of participants employed in this study (i.e., amateur softball pitchers) did not allow for comparison with professional athletes. Regarding the pitching accuracy, a previous study has reported that the strike rates in 480 regular season Chinese Professional Baseball League games were approximately 50% [6]. In addition, intercollegiate baseball pitchers from University Baseball League Division I in Taiwan had approximately 45% of strike rates during the simulated baseball games [21]. These rates are consistent with the present result. Because ball velocity and pitching accuracy may interact during actual softball games, and pitching accuracy in professional baseball pitchers was significantly higher than in amateur baseball pitchers [36], further research is needed to investigate the effect of ice slurry ingestion on pitching performance during professional softball games. Second, we did not perform ambient-temperature fluid trials or no-ingestion trials. However, cold fluid ingestion is a common practice among many athletes [19]. Third, the pitching accuracy was subjectively determined by an investigator. Although a single umpire is responsible for calling balls and strikes in softball games, the lack of objective determination of the strike zone and whether the ball passed the strike zone could lead to human error.

## Conclusions

This study demonstrated that ice slurry ingestion before (5.0 g·kg^−1^) and between (1.25 g·kg^−1^) innings improved thermal (*T*_re_), cardiovascular (HR), and perceptual (RPE, TS, and TC) strain compared with cool fluid ingestion. However, this strategy did not affect pitching performance.

## Supplementary Information

**Additional file 1:** **Supplementary Table 1.** Mean data in for all participants between trials (mean ± standard deviation, *n* = 7). 

**Additional file 2: Supplementary Table 2.** Mean data of male participants between trials (mean ± standard deviation, *n* = 4).

**Additional file 3: Supplementary Table 3.** Mean data of female participants between trials (mean ± standard deviation, *n* = 3).

## Data Availability

The datasets during and/or analyzed during the current study are available from the corresponding author on reasonable request.

## Abbreviations

CON: Control trial
HR: Heart rate
ICE: Ice trial
REST: Seated rest in a dugout for 15 min
RPE: Rate of perceived exertion
*T*_c_: Body core temperature
TC: Thermal comfort
*T*_re_: Rectal temperature
TS: Thermal sensation
$\overline{T }$_sk_: Mean skin temperature
*Δ*: Change
